# Allergic rhinitis in northern vietnam: increased risk of urban living according to a large population survey

**DOI:** 10.1186/2045-7022-1-7

**Published:** 2011-08-11

**Authors:** Hoàng Thị Lâm, Nguyễn Văn Tường, Linda Ekerljung, Eva Rönmark, Bo Lundbäck

**Affiliations:** 1 Unit of Lung & Allergy Research, National Institute of Environmental Medicine, IMM, Karolinska Institutet, Stockholm, Sweden; 2 Department of Allergy, Hanoi Medical University, Hanoi, Vietnam; 3 Department of Allergy and Clinical Immunology, Bachmai Hospital, Hanoi, Vietnam; 4 Department of Scientific Research, Hanoi Medical University, Hanoi, Vietnam; 5 Krefting Research Centre, Institute of Medicine at Sahlgrenska Academy, University of Gothenburg, Gothenburg, Sweden; 6 The OLIN Studies, Dept of Medicine, Sunderby Hospital of Norrbotten, Luleå, Sweden; 7 Department of Public Health and Clinical Medicine, University of Umeå, Umeå, Sweden

**Keywords:** Allergic rhinitis, nasal blocking, runny nose/chronic rhinitis, epidemiology, Vietnam

## Abstract

**Background:**

Little is known about prevalence and risk factors of allergic rhinitis and chronic nasal symptoms among adults in Vietnam. We aimed to estimate the prevalence, risk factor patterns and co-morbidities of allergic rhinitis and chronic nasal symptoms in one urban and one rural area in northern Vietnam.

**Methods:**

A cross-sectional questionnaire survey was conducted from August 2007 to January 2008 in urban Hoankiem and rural Bavi in Hanoi among adults aged 21-70 years. Of 7008 randomly selected subjects, 91.7% participated in Bavi and 70.3% in Hoankiem.

**Results:**

Allergic rhinitis ever or chronic nasal symptoms were reported by 50.2%. The prevalence of allergic rhinitis ever was considerably higher in Hoankiem compared to Bavi, 29.6% vs 10.0% (p < 0.001). Allergic rhinitis ever and chronic nasal symptoms were both significantly associated with asthma and respiratory symptoms, respectively (p < 0.001). Exposure to gas, dust or fumes at work was significantly associated with allergic rhinitis ever, OR 1.57 (95% CI 1.34 - 1.84), nasal blocking, OR 1.90 (95% CI 1.68 - 2.15) and runny nose, OR 1.32 (95% CI 1.17 - 1.49), while somewhat surprisingly no association with smoking was found. Female sex was a significant risk factor for both nasal blocking and runny nose.

**Conclusions:**

Allergic rhinitis ever was considerably more common in the urban area. Nasal blocking and runny nose was each reported by about one third of the studied sample with no major urban-rural difference. Further, exposure to air pollution at work was significantly associated with allergic rhinitis ever, nasal blocking and runny nose.

## Introduction

The prevalence of allergic diseases including allergic rhinitis has been increasing all over the world since about the middle of the past century [1]. The prevalence of allergic rhinitis differs between countries and even between areas within countries [2]. These differences may partly be due to different definitions and methods used [3]. Recent epidemiological studies suggest that urban living and exposure to air pollution at home and at work place are risk factors for allergic rhinitis [4], while rural living [5], particularly living on a farm the first year of life may have a protective effect on allergic rhinitis [6]. The most common cause of allergic rhinitis is allergic sensitization to airborne allergens [7].

Allergic rhinitis is not a cause of mortality, but it is a burden in the society since it has a major impact on quality of life and daily functioning [8]. The disease is intimately associated with asthma, a major public health problem in many countries [9]. Total asthma control may be difficult to reach if treatment of the concomitant rhinitis is not addressed [10].

The prevalence of allergic rhinitis in European countries amounts to 20-30% [6,11,12]. In south-east Asia there are only few published studies of allergic rhinitis among adults [13,14]. Chronic rhinitis is even more sparsely studied, and there are no data from adults in south-east Asia including Vietnam about nasal congestion or chronic rhinitis. Among children in Thailand more than 40% had rhinitis according to the International Study of Asthma and Allergies in Children (ISAAC) study protocol [15], while it was 11% among children in Hanoi, Vietnam [16].

Because the lack of data from adults, we performed a population study in urban and rural northern Vietnam with the aims to: (i) estimate the prevalence of allergic rhinitis ever, chronic nasal blocking and chronically runny nose, (ii) explore the associations between each of the rhinitis conditions with other respiratory symptoms, and (iii) evaluate the risk factors for each of the rhinitis conditions.

## Materials and methods

### Study area

The study was performed in two areas of Hanoi in northern Vietnam. The urban Hoankiem is an inner-city district and comprises the biggest trading center of Hanoi and had in 2007 a high population density of 32 703 inhabitants/km^2^. The rural Bavi is a typical rural village 60 km west of central Hanoi with agricultural production and livestock breeding as the main economic activities.

### Study population

The sample was randomly chosen from the population register in both areas. The age distribution was 21-70 years old (born 1937 to 1986). Of 108 000 subjects in Hoankiem, 4000 were selected. However, 992 subjects were excluded because the addresses could not be traced. In Bavi, 4000 subjects of totally 51 000 were chosen. In total, the sample thus included 7008 subjects. The questionnaire was completed by 5872 subjects; in Bavi by 3667 (91.7%) subjects and in Hoankiem by 2115 (70.3%), p < 0.001. The participation rate was similar by age and gender. A pilot study, the study area and population, methods and the participation rate have previously been described in detail [17], as well as the field survey unit of Bavi [18]. This study was approved by the Medicine Ethics Research Committee of Hanoi Medical University in July 2006.

### Questionnaire

The questionnaire was taken from the FinEsS questionnaire [19], which was modified from the Swedish OLIN study questionnaire [20]. It has been validated by studies performed in Sweden, Finland and Estonia [19-22] and will be clinically validated in Vietnam. The questionnaire included 25 questions about respiratory symptoms, diagnoses, smoking habits, occupation and exposure to dust, gas and fumes at work places. The study was conducted from August 2007 to January 2008.

### Definitions

#### Allergic rhinitis ever

Do you have or have you ever had allergic rhinitis?

#### Nasal blocking

Do you have blocking of your nose more or less permanently?

#### Runny nose

Do you have rhinitis or a runny nose more or less permanently?

The wording of respiratory symptoms, asthma, smoking habits, farm living, occupation and occupational exposure has previously been described [17].

### Analysis

The SPSS version 16.0 was used for statistical analysis. Difference in prevalence was calculated using χ2 test and a p-value < 0.05 was considered statistically significant. Risk factors for allergic rhinitis, chronic rhinitis and nasal blocking, respectively, were calculated by using multiple logistic regression analysis. Demographic and exposure data were used as independent variables. They included sex, age, area, smoking habits, occupational exposure to dust, gases and fumes, and being raised on a farm during the first year of life. The results are expressed as odds ratios (OR) with 95% confidence intervals (CI).

## Results

### Prevalence of rhinitis

The prevalence of allergic rhinitis ever was considerably higher in Hoankiem compared to Bavi, 29.6% vs. 10.0% (p < 0.001). Moreover, allergic rhinitis ever was equally common in men and women and without significant difference by age. Nasal blocking was also more common in Hoankiem than Bavi, 40.2% vs. 31.0% (p < 0.001). The prevalence of runny nose, the most common nasal condition, was equally common in the two areas: Hoankiem 39.0% and Bavi 39.4% (Table 1). Both nasal blocking and runny nose was equally common in the two age groups of subjects aged 21-45 years and 46-70 years. More women than men had nasal blocking (p < 0.001) and runny nose (p < 0.001), respectively, in both areas.

**Table 1:** Prevalence of allergic rhinitis ever, nasal blocking and runny nose symptoms by age, sex and area.

Symptom or condition		21-45 y		46-70 y		All	Difference by (p -value)		
				
		M	W	M	W		Sex	Age	Area
**Allergic rhinitis ever**	**Hoankiem**	29.0	28.9	29.3	31.7	29.6	0.605	0.443	< 0.001
	**Bavi**	9.0	10.0	10.6	11.0	10.0	0.422	0.209	
**Nasal blocking**	**Hoankiem**	38.4	39.4	40.1	43.7	40.2	0.324	0.167	< 0.001
	**Bavi**	26.7	36.2	27.5	32.3	31.0	< 0.001	0.293	
**Runny nose**	**Hoankiem**	36.1	40.1	39.5	40.7	39.0	0.217	0.360	0.754
	**Bavi**	35.0	43.4	38.0	41.3	39.4	< 0.001	0.806	

Difference (p-value) by sex, age and area.

Similar to the difference in prevalence of allergic rhinitis ever between Bavi and Hoankiem, living on a farm during the first years of life, compared to those who had not, yielded a considerably lower prevalence of allergic rhinitis ever (Table 2). This was true among both men and women irrespectively of age. A similar difference but of lower magnitude was found for nasal blocking, while for runny nose no obvious difference was found for living or not living on a farm during the first year of life (Table 2).

**Table 2:** Prevalence of allergic rhinitis ever, nasal blocking and runny nose in men, women and age-groups by childhood farm living.

**Symptom or condition**	**Men**			**Women**			**21-45 y**			**46-70 y**		
	
	**Farm living**		**P-value**	**Farm living**		**P-value**	**Farm living**		**P-value**	**Farm living**		**P-value**
	**Yes**	**No**		**Yes**	**No**		**Yes**	**No**		**Yes**	**No**	
	
**Allergic rhinitis ever**	11.1	27.7	< 0.001	11.6	28.9	< 0.001	10.6	26.7	< 0.001	12.7	30.5	< 0.001
**Nasal blocking**	28.9	36.6	< 0.001	35.7	40.0	0.022	32.8	36.5	0.030	32.8	40.9	< 0.001
**Runny nose**	37.2	35.7	0.426	43.1	39.4	0.058	40.1	36.5	0.039	40.1	39.1	0.551

Difference (p-value) by childhood farm living and no childhood farm living in men, women and by age group. *Yes *indicates childhood farm living. *No *indicates no childhood farm living.

**Figure 1:**
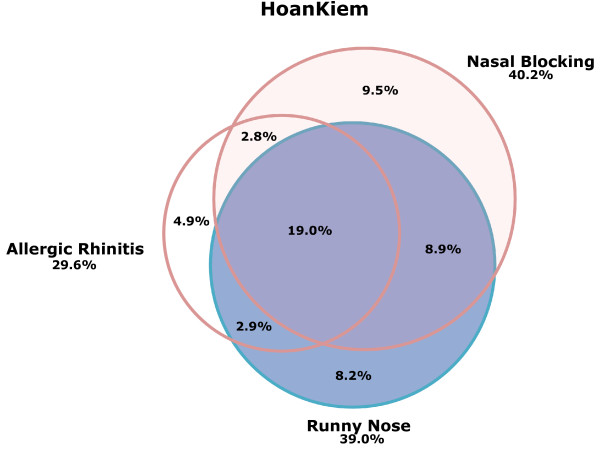
**Venn-diagram of prevalence of allergic rhinitis ever, nasal blocking and a runny nose in Hoankiem**.

**Figure 2:**
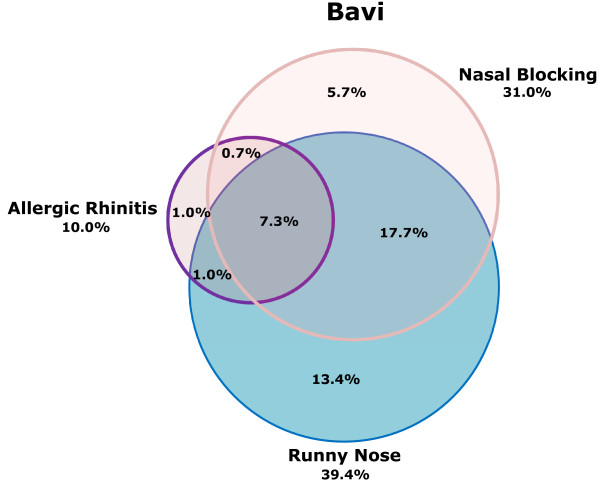
**Venn-diagram of prevalence of allergic rhinitis ever, nasal blocking and a runny nose in Bavi**.

Of the whole study sample, 11.6% had all the three nasal conditions, i.e. allergic rhinitis ever, nasal blocking and runny nose. Further, 26.0% had both runny nose and nasal blocking, 13.0% had both nasal blocking and allergic rhinitis ever, while 13.3% had both runny nose and allergic rhinitis ever. Thus, the majority of those having allergic rhinitis ever also had any of the two chronic nasal conditions. The prevalence of having any of the three nasal conditions was reported by 50.2% of the whole study sample. A Venn-diagram illustrates the associations between allergic rhinitis ever, nasal blocking and runny nose in Hoankiem and Bavi, respectively (Figure 1) Figure 2.

### Rhinitis and respiratory symptoms

Among the subjects reporting allergic rhinitis ever, nasal blocking and runny nose, the prevalence of physician-diagnosed asthma and respiratory symptoms were considerably greater (p < 0.001 for all comparisons) compared with those not having the nasal conditions (Table 3). Physician-diagnosed asthma and symptoms common in asthma were higher among those who had allergic rhinitis ever compared to those who had not: physician-diagnosed asthma 10.4%; any wheeze 9.3%; recurrent wheeze 8.9% vs about 3% for all these conditions among those not having allergic rhinitis ever (Table 3). The proportion of longstanding cough and sputum production was equally common among the subjects having each of the three nasal conditions (Table 3).

**Table 3:** Prevalence of physician-diagnosed asthma and respiratory symptoms in the whole sample and among the subjects reporting allergic rhinitis ever, nasal blocking and runny nose.

**Symptoms or conditions**	**Whole sample**	**Allergic rhinitis ever**		**P-value**	**Nasal blocking**		**P-value**	**Runny nose**		**P-value**
		
		**Yes**	**No**		**Yes**	**No**		**Yes**	**No**	
	
**Physician diagnosed asthma**	**3.9**	10.4	2.5	< 0.001	7.4	2.0	< 0.001	5.8	2.6	< 0.001
**Last 12 months wheeze**	**4.2**	9.3	3.2	< 0.001	7.6	2.4	< 0.001	7.2	2.3	< 0.001
**Recurrent wheeze**	**3.9**	8.9	2.6	< 0.001	7.1	1.9	< 0.001	6.6	1.8	< 0.001
**Long standing cough**	**15.9**	24.3	14.1	< 0.001	28.8	9.0	< 0.001	24.6	10.2	< 0.001
**Sputum production**	**13.9**	21.8	12.3	< 0.001	21.8	9.7	< 0.001	20.4	9.7	< 0.001

Difference (p-value) for each symptom between those with and without each nasal condition

### Multivariate relationships

Living in Hoankiem vs living in Bavi was significantly associated with allergic rhinitis ever, OR 3.94 (95% CI 3.40-4.56), and nasal blocking, OR 1.62 (95% CI 1.44-1.81), while living in Hoankiem was not a significant risk factor for runny nose (Table 4). Female sex was a significant risk factor for nasal blocking and runny nose, but not for allergic rhinitis ever. Exposure to gas, dust or fumes at work place was significantly associated with all three nasal conditions. Neither current nor ex-smoking, or age group, was significantly associated with any of the three nasal conditions (Table 4). Living the first year of life on a farm did not further increase the risk of urban domicile for any of the nasal condition, and was thus not included in the final multivariate model.

**Table 4:** Risk factors for allergic rhinitis ever, nasal blocking and runny nose by multiple logistic regression analysis.

Independent variables		Dependent variables		
**Variables**	**Categories**	**Allergic rhinitis ever**	**Nasal blocking**	**Runny nose**
		**OR (95% CI)**	**OR (95% CI)**	**OR (95% CI)**

**Sex**	**Women**	1.00 (0.84-1.21)	1.47 (1.26 - 1.72)	1.36 (1.18 - 1.58)
**Age**	**≥ 45**	1.11 (0.96 - 1.29)	1.03 (0.92 - 1.16)	1.05 (0.94 - 1.18)
**Areas**	**Hoankiem**	3.94 (3.40 - 4.56)	1.62 (1.44 - 1.81)	1.01 (0.90 - 1.13)
**Exposed to gas, dust, fumes**	**Yes**	1.57 (1.34 - 1.84)	1.90 (1.68 - 2.15)	1.32 (1.17 - 1.49)
**Smoking**	**Non-smoker**	1	1	1
	**Ex-smoker**	1.20 (0.77 - 1.85)	0.93 (0.64-1.34)	0.98 (0.70 - 1.38)
	**Current- smoker**	0.81 (0.66 - 1.00)	1.13 (0.96 - 1.34)	1.13 (0.96 - 1.32)

Risks in odd ratios (OR) with 95% confidence intervals (CI).

## Discussion

Our main finding was the high prevalence of allergic rhinitis ever in urban Hoankiem, 30%, in contrast to 10% in Bavi, and a difference of similar magnitude was found between those who had lived on a farm during the first year of life compared with those who had not. The other important finding was the very high prevalence, 30-40%, of both chronic nasal blocking and chronically runny nose in both urban Hoankiem and rural Bavi.

For comparison, there are no published data about the prevalence of chronic nasal conditions or symptoms from south-east Asia. These symptoms have been sparsely studied also in westernized countries, and there are only a few publications in the literature [23-27]. In contrast to westernized countries [10,25], the chronic nasal conditions, particularly runny nose, did not increase significantly by age in our study. In a recent European study, runny nose was reported by 20% in the UK [24]. A recent study from the western part of Sweden found the prevalence of nasal blocking to be 15% and of chronically runny nose to be 13% [27]. We thus conclude chronic nasal symptoms to be much more common in Vietnam.

Our results suggest the prevalence of allergic rhinitis in Hanoi to be similar or even greater than has been reported from European countries. A pan-European study found the average prevalence of allergic rhinitis to be 23%, with a variation ranging from 17% for Italy to 29% for Belgium [11], the latter on a similar magnitude as in a recent Swedish study [11]. When comparing with results from studies among adults in East-Asia, the prevalence of allergic rhinitis in Hanoi remains high. A questionnaire study in large cities of China based on telephone interviews in 2004-2005 found a prevalence of allergic rhinitis ranging from 9 to 24% [13]. In South-Korea an even lower prevalence of allergic rhinitis was found, 6-10% [14]. The protocol of the International Study of Asthma and Allergies in Children (ISAAC) has been used in some studies of children and 13-14 years old teenagers. In Thailand the prevalence of rhinitis based on the written ISAAC questionnaire increased from 33% in 1995 to 43% in 2003 [15]. Among children in urban Hanoi, the prevalence of doctors' diagnosed allergic rhinitis was 11% in 2001[16]. This was somewhat lower than found in studies following the ISAAC protocol in Singapore, Taiwan and Malaysia [28-30].

There are several possible explanations to the reported high prevalence of ever having had allergic rhinitis in urban Hanoi. The population has rapidly increased accompanied by a rapid increase in traffic. Air pollution can increase the allergenic potency and thereby promote sensitization and an exaggerated response to allergens in nasal airways [31]. In urban Hanoi, people use considerably more private than public transports, and the majority use motorbikes. The high population density and narrow roads with traffic jams every day contribute to heavy air pollution in the city. Air pollution is a causal factor to damage of the nasal mucosa [32]. Moreover, poor indoor environment with a very high humidity, moulds, house dust mites, cockroaches and several types of animal dander may add to or exacerbate health inequalities resulting from air pollution [33].

The low prevalence of allergic rhinitis ever in the rural area is in line with findings from westernized countries [6,34,35]. However, the very large urban rural difference in our study may have several contributing explanations. The prevalence of chronic nasal symptoms was very high also in Bavi. The high prevalence of chronic nasal symptoms may reflect an underestimation of allergic rhinitis in the rural area, and many individuals probably do not recognize their nasal symptoms as symptoms of allergy. Chronic nasal symptoms may occur in allergic rhinitis and in other types of upper airway diseases such as nasal polyposis, rhino-sinusitis and bacterial infections.

All lower respiratory symptoms and asthma were significantly more common among the subjects with the nasal conditions, results in line with previous findings [10,27]. In line with what was expected, this association was most pronounced between asthma and allergic rhinitis ever [10,27]. Epidemiologic studies throughout the world have consistently shown that asthma and rhinitis often coexist [36]. In a Spanish study, 49% of the patients with allergic rhinitis had concomitant asthma [37]. Further, the vast majority of patients with asthma have rhinitis [10,27]. It might be said that asthma and allergic rhinitis are different manifestations of the same disease, and the concept "united airways" has been proposed [38].

The risk factor analysis verified urban living to be the dominating risk factor for ever having had allergic rhinitis. This was the case also for nasal blocking but not for runny nose. Another important risk factor for each of the three nasal conditions was occupational exposure from dust, gases and fumes, results in line with several European studies [4,27,39]. Most studies have not found smoking to be a risk factor for allergic rhinitis among adults, while other studies have found smoking to be strongly associated with chronic nasal symptoms [26,27]. Surprisingly, in our study we did not found this association. One reason can be that also non-smokers are more or less continuously exposed to tobacco smoke due to the very high smoking prevalence among men in Vietnam [17].

We further found that being a woman was a risk factor for nasal blocking and runny nose. In Vietnam women are responsible for house works, especially for preparing food. The use of solid fuels in poorly ventilated homes results in high levels of indoor air pollution. Randomized controlled trials have shown that women who used biomass stove or chimney woodstove most of the time, compared with those using traditional indoor open fire, were at a lower risk of developing respiratory symptoms [40,41].

There are several strengths with our study. The randomly selected study sample with a high participation rate, similar to studies in northern Europe [19-22], support both the representativeness of the participants. As the symptom distribution in most aspects is similar to that in Europe, the use of a validated questionnaire also contributes to the validity of our results. There are, however, also weaknesses with our study. First, there may be a difference between the study areas about the understanding of diagnoses and diseases, which may have caused bias on disease level, however probably not on symptom level. Another weakness is general for questionnaire studies, i.e. the lack of objective clinical measurements such as IgE. One of our studies in progress aims to identify relevant sensitization profiles among adults in northern Vietnam. So far a pilot study shows that similar and large portions in both Hoankiem and Bavi of skin prick tested subjects among those reporting allergic rhinitis are sensitized to common airborne allergens, mainly mites.

In conclusion, the prevalence of chronic nasal conditions was very high, about 30-40% in both men and women irrespectively of age. Exposure to household smoke from open fires might have contributed to a higher prevalence among women. The prevalence ever having had of allergic rhinitis in Hanoi was 30%, while it was only 10% in the rural area. Exposure from dust, gases and fumes at work places was significantly associated with all rhinitis conditions.

## Competing interests

The authors declare that they have no competing interests.

## Authors' contributions

HTL contributed to the design of the study and performed the study, participated in analysing and interpreting of data, wrote and revised the article for important intellectual content and approved the version to be published. NVT contributed to the design of the study, participated in analysing and interpreting of data and approved the version to be published. LE participated in analysing and interpreting of data, participated in writing and in the revision of the article for important intellectual content and approved the version to be published. ER contributed to the design of the study, participated in analysing and interpreting of data, wrote and revised the article for important intellectual content and approved the version to be published. BL contributed to design the study, participated in analysing and interpreting of data, wrote and revised the article for important intellectual content and approved the version to be published.

All authors read and approved the final manuscript.
